# Effect of Spontaneous and Water-Based Passivation on Components and Parameters of Ti6Al4V (ELI Grade) Surface Tension and Its Wettability by an Aqueous Solution of Sucrose Ester Surfactants

**DOI:** 10.3390/molecules27010179

**Published:** 2021-12-28

**Authors:** Joanna Krawczyk, Amparo María Gallardo-Moreno, María Luisa González-Martín

**Affiliations:** 1 Department of Interfacial Phenomena, Institute of Chemical Sciences, Faculty of Chemistry, Maria Curie-Skłodowska University, 20-031 Lublin, Poland; 2 Department of Applied Physics, Faculty of Science, Extremadura University, Avda. de Elvas, s/n, 06006 Badajoz, Spain; amparogm@unex.es (A.M.G.-M.); mlglez@unex.es (M.L.G.-M.); 3 Networking Research Center on Bioengineering, Biomaterials and Nanomedicine (CIBER-BBN), 06006 Badajoz, Spain; 4 University Institute of Extremadura Sanitary Research (INUBE), 06006 Badajoz, Spain

**Keywords:** biomaterials, wettability, sucrose fatty acid esters, contact angle, components and parameters of solid surface tension

## Abstract

Solid wettability is especially important for biomaterials and implants in the context of microbial adhesion to their surfaces. This adhesion can be inhibited by changes in biomaterial surface roughness and/or its hydrophilic–hydrophobic balance. The surface hydrophilic–hydrophobic balance can be changed by the specifics of the surface treatment (proper conditions of surface preparation) or adsorption of different substances. From the practical point of view, in systems that include biomaterials and implants, the adsorption of compounds characterized by bacteriostatic or bactericidal properties is especially desirable. Substances that are able to change the surface properties of a given solid as a result of their adsorption and possess at least bacteriostatic properties include sucrose ester surfactants. Thus, in our studies the analysis of a specific surface treatment effect (proper passivation conditions) on a biomaterial alloy’s (Ti6Al4V ELI, Grade 23) properties was performed based on measurements of the contact angles of water, formamide and diiodomethane. In addition, the changes in the studied solid surface’s properties resulting from the sucrose monodecanoate (SMD) and sucrose monolaurate (SML) molecules’ adsorption at the solid–water interface were also analyzed. For the analysis, the values of the contact angles of aqueous solutions of SMD and SML were measured at 293 K, and the surface tensions of the aqueous solutions of studied surfactants measured earlier were tested. From the above-mentioned tests, it was found that water environment significantly influences the components and parameters of Ti6Al4V ELI’s surface tension. It also occurred that the addition of both SMD and SML to water (separately) caused a drop in the water contact angle on Ti6Al4V ELI’s surface. However, the sucrose monolaurate surfactant is characterized by a slightly better tendency towards adsorption at the solid–water interface in the studied system compared to sucrose monodecanoate. Additionally, based on the components and parameters of Ti6Al4V ELI’s surface tension calculated from the proper values of components and parameters of model liquids, it was possible to predict the wettability of Ti6Al4V ELI using the aqueous solutions of SMD and SML at various concentrations in the solution.

## 1. Introduction

Metallic implants are widely used for biomedical applications. The most common are used in orthopedic surgery or dental applications, where they serve as temporary or permanent implants in the body. Metals possess many properties which make them desirable for bone repair, however, their corrosion stability and biocompatibility can be problems. The body contains many compounds, for example, water, sodium chloride and proteins, which are very reactive to metals. Since metals possess the ability to alloy, metals can be modified by using elements that do not exert adverse effects in the body. For example, titanium alloys are able to tolerate the corrosive environment of the body to a great extent [1,2].

Titanium is one of the most common elements used in the area of metallic implants. This is because of its excellent combination of mechanical properties, corrosion resistance and biocompatibility. Its properties can be widely modified by varying the alloying elements (for example Zr, Ta, Nb, Mo, Al and V) and volume atomic composition (O, C, Fe) [1]. Commercially pure titanium (Ti) and Ti6Al4V extra low interstitial alloy (Ti6Al4V ELI, Grade 23) are the most used materials for implants in biomedical applications, especially orthopedic ones [1]. In addition, due to the fact that titanium is very reactive upon exposure to air, an oxide layer is formed on its surface almost immediately (passivation process). The passivation layer on titanium’s surface prevents the transfer of ions from the implant to the bodily fluids, and in this way influences its corrosion resistance and biocompatibility [1,2]. It also affects the bactericidal properties of the implant. The oxide layer’s properties (composition, thickness, homogeneity, stability) depend mainly on the titanium surface preparation (for example polishing, manufacturing, etc.) [3] and the conditions of the passivation layer’s formation [3,4]. In turn, these properties are reflected in the components and parameters of the titanium’s surface tension [5]. However, it is also known that the corrosion resistance of titanium alloys can be changed by, for example, the presence of fluoride (in the case of dental implants) [1,6]. Thus, it should be remembered that there is no material which is completely bioinert. In the case of dental implants, the probability of implant damage is around 5–20% [1]. For these reasons, the literature reports numerous methods for titanium and titanium alloy surface modifications. These modifications are aimed at improving the stability of the passivation layer (corrosion resistance) or preventing bacterial attachment to the implant [1,3,4,6,7,8,9,10,11]. Stability of the passivation layer on the titanium or titanium alloy’s surface can be changed by changing the conditions of its formation and preparation (environment, temperature, drying method, polishing, etc.). For example, the thermal oxidation (at a suitably high temperature) method is widely used to improve the corrosion and wear resistance of Ti alloys [1,7,8,11]. This results from the fact that the rutile TiO_2_ layer covering the titanium alloy is characterized by greater bonding strength and thickness as compared to the naturally formed oxide. This is especially important for dental and orthopedic implant applications [6,9]. On the other hand, it is essential to obtain good reproducibility of a given implant material’s properties after its modification. These properties are particularly related to the biomaterial wettability and its surface free energy.

Surface modifications related to bacterial attachment to the implant surface can include the surface roughness or wettability changes. For this purpose, different techniques and different substances are used [1,2,4,12]. The substances used for implant surface modifications (for example, coatings) are at least bacteriostatic and biocompatible with us [4]. Such properties are possessed by the substances which belong to one of the sugar surfactant groups—that is, sucrose fatty acid esters (SE) [13,14,15]. Due to their ability to adsorb at different interfaces, they can influence the surface properties of the solid and those of the solid–liquid interface by changing the solid wettability. Considering the fact that adhesion of bacteria to a given solid depends largely on its wettability, this process seems to be decisive.

Thus, the purpose of our paper was to determine the relationships among the adsorption, adhesion and wetting properties of sucrose capric acid ester (sucrose monodecanoate) (SMD) and sucrose lauric acid ester (sucrose monolaurate) (SML) (separately) in Ti6Al4V ELI–aqueous solution of surfactant–air systems. In addition, the influences of Ti6Al4V ELI surface preparation conditions on its hydrophobic–hydrophilic balance were determined. Next, the obtained results were used for wettability and adhesion process prediction for aqueous solutions of SMD and SML within the Ti6Al4V ELI–aqueous solution of surfactant–air system.

## 2. Results and Discussion

### 2.1. Components and Parameters of Ti6Al4V-ELI’s Surface Tension

The surface properties of a given solid play a crucial role in many processes occurring at different interfaces and in different fields (industry, medicine and daily life). Among them, wetting plays a very important role, especially in the solid–liquid (water)–air systems. Thus, to describe properly the solid wettability process in a given system, its surface properties should be properly described.

In systems including solid, liquid and gas phases, wetting of a given solid (expressed by the contact angle value (θ)) depends on the properties of the solid and liquid, and the kinds of intermolecular interactions in the liquid and solid. The dependence can be expressed by the Young equation [16]:

(1) $$\gamma_{S}-\gamma_{SL}=\gamma_{L}\cos\theta$$

where $\gamma_{S}$ is the solid surface tension, $\gamma_{L}$ is the liquid surface tension and $\gamma_{SL}$ is the solid–liquid interface tension.

To solve Equation (1) and to describe the wetting process in a given solid–liquid–air system properly, the values of solid and liquid surface tension and solid–liquid interface tension must be known. The values of $\gamma_{L}$ and θ can be directly measured using various methods [17,18]. However, determination of solid surface tension or solid–liquid interface tension comes across some difficulties. The indirect method based on the measurements of θ in the given system is most frequently applied for determination of surface tension of a solid and solid–liquid interface tension. To determine the solid surface tension, the measured values of contact angle for model liquids on the solid surface and proper approaches to the solid–liquid interface tension must be used. Among them, the approaches proposed by van Oss et al. [19,20,21,22], Owens and Wendt [23] and Neuman et al. [24,25,26] are the most frequently applied. In this paper, to determine Ti6Al4V ELI’s surface tension, the van Oss et al. approaches to the liquid–liquid and solid–liquid interface tensions were used.

Van Oss et al. proposed the following equation for the solid–liquid interface tension [19,20,21,22]:

(2) $$\gamma_{SL}=\gamma_{S}+\gamma_{L}-2\sqrt{\gamma_{S}^{LW}\gamma_{L}^{LW}}-2\sqrt{\gamma_{S}^{+}\gamma_{L}^{-}\;}-2\sqrt{\gamma_{S}^{-}\gamma_{L}^{+}}$$

where $\gamma_{L}^{LW}$ and $\gamma_{S}^{LW}$ are the Lifshitz–van der Waals components of the liquid and solid surface tension; $\gamma_{L}^{+}$ and $\gamma_{S}^{+}$ are the electron-acceptor parameters of the acid-base component of the liquid ($\gamma_{L}^{AB}$) and solid ($\gamma_{S}^{AB}$) surface tension; and $\gamma_{L}^{-}$ and $\gamma_{S}^{-}$ are the electron-donor parameters of the acid-base component of the liquid and solid surface tension, respectively.

Taking into account Equations (1) and (2), it is possible to write [22]:

(3) $$\gamma_{L}\left(\cos\theta +1\right)=2\left(\sqrt{\gamma_{L}^{LW}\gamma_{S}^{LW}}+\sqrt{\gamma_{L}^{+}\gamma_{S}^{-}}+\sqrt{\gamma_{L}^{-}\gamma_{S}^{+}}\right)$$

In the case of solids whose surface tension results only from the Lifshitz–van der Waals intermolecular interactions, Equation (3) has the form [22]:

(4) $$\gamma_{L}\left(\cos\theta +1\right)=2\sqrt{\gamma_{L}^{LW}\gamma_{S}^{LW}}$$

As follows from Equations (3) and (4), to determine the solid surface tension from the van Oss et al. approaches, despite the exact model liquid’s surface tension, its components and parameters must also be known. It is possible to solve the van Oss et al. equation for the components and parameters of solid surface tension in two different ways. The first one is based on the application of the contact angle values of three polar liquids, and the other one is based on the values of the contact angle for two polar liquids and one apolar liquid [27]. In our studies, the latter method was applied. Before the contact angle measurements on Ti6Al4V ELI’s surface, atomic composition analysis was performed. This is especially important in the case of implants used in medicine, as the alloying elements and impurity constituents could influence an implant’s interactions with the surrounding environment, and could lead to the formation of defective passive layers on titanium alloys [1,8,28]. The atomic composition of Ti6Al4V ELI given by the manufacturer was confirmed by the XRF analysis. The obtained results (Table 1) align with the specification sheet data supplied by the manufacturer (according to ASTM F 136-02a). They also confirm the purity grade of the studied implant (Grade 23) in relation to the main elements included in the alloy. After the XRF analysis, the studied alloy discs were prepared in a proper way (procedure *A*, *B* or *C*), and the advancing contact angles of three model liquids—two polar and one apolar (water, formamide and diiodomethane)—were measured. The obtained contact angle values are presented in Table 2. Taking into account the measured contact angle values and the model liquids’ surface tensions and their components and parameters (Table 3) [19,20,21,22], based on Equation (3), the values of Ti6Al4V ELI surface tension and its components and parameters were calculated. The components and parameters of the model liquids’ surface tension calculated on the basis of the contact angle measurements (1) or deduced from the liquid–liquid interface tension (2), were taken from the literature and summarized in Table 3 [19,20,21,22,29,30].

The components and parameters of the model liquids’ surface tension determined from the liquid—liquid interface tension [19,20,21,22] were displayed to show their influences on the components and parameters of solid surface tension in the studied system. The calculated surface tension of Ti6Al4V ELI and its components and parameters values are also presented in Table 3. As shown in Table 2, the water, formamide and diiodomethane contact angle values on Ti6Al4V ELI’s surface depend largely on the conditions of the surface preparation. In general, the passivation of Ti6Al4V ELI in the water environment caused significant growth in the contact angle values of the studied model liquids, which reflect great changes in Ti6Al4V ELI’s surface properties. Similar observations were also made earlier for the water contact angle [31]. This indicates the temperature and water environment impact the titanium alloy’s surface properties. As shown in Table 2, the contact angle of water measured on Ti6Al4V ELI is consistent with the literature data [8,32]. The minimal values of water contact angle taken from the literature (40°) [31] are close to those measured in the paper on Ti6Al4V ELI’s surface, which was prepared according to procedure *A* (43 ± 5°) (Table 2). It should be stated also that the source (manufacturer) of the Ti6Al4V disc could influence the average contact angle value of a given liquid. Table 2 shows that the standard deviations of the measured contact angle values of model liquids on Ti6Al4V ELI’s surface prepared according to procedure *C* are smaller than those measured on Ti6Al4V ELI’s surface prepared according to the procedure *A* or *B.* They also slightly depend on the kind of model liquid. This indicates that the specific surface treatment causes the oxide layer which is formed on Ti6Al4V ELI’s surface to be more homogeneous.

This is of importance regarding the corrosion resistance of the studied alloy. Based on the measured contact angle values, it was also stated that passivation (in the water environment) and drying (in both oven and desiccator) time did not influence the contact angle values of the model liquids. It was also noticed that rubbing (procedure *C*) of the titanium alloy surface with a cotton material under hot water (before the cleaning procedure) caused an increase in average water contact angle value (from 75° to 81°), and that the standard deviations of all measured contact angle values of model liquids diminished (Table 2). The above-mentioned considerations could be also reflected in the surface characteristics.

**Figure 1:**
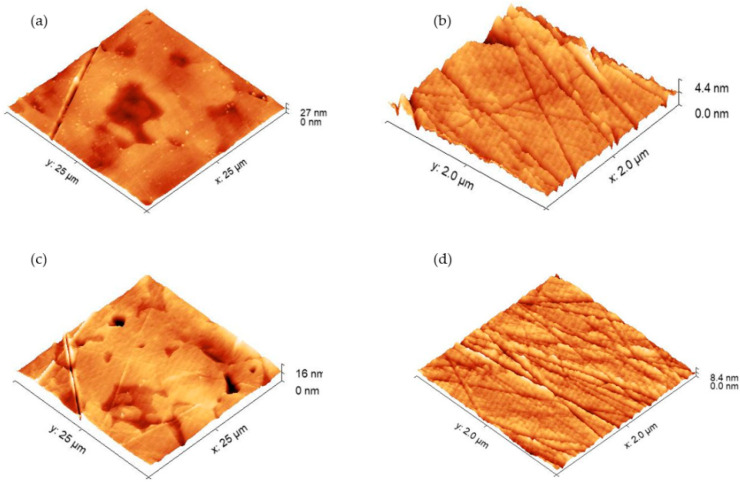
AFM topographical images of Ti6Al4V ELI’s surface prepared according to procedure *B* ((**a**,**b**) scanning scale equal to 25 × 25 µm^2^ and 2 × 2 µm^2^, respectively) and procedure *C* ((**c**,**d**) scanning scale equal to 25 × 25 µm^2^ and 2 × 2 µm^2^, respectively).

The topographic surface characterization of Ti6Al4V ELI was performed through atomic force microscopy. For this purpose, the titanium alloy surfaces were prepared according to the described procedures. It was interesting to get to discover if there are any differences in surface characteristics which could be associated with the surface preparation procedure (*B* or *C*). The obtained results are presented in Figure 1. In every case, we can see a texture typically associated with a mirror-polished metal surface: very smooth and with crisscrossed straight lines that come from the polishing process. The 25 × 25 µm^2^ images also reflect the biphasic microstructure of the alloy: phase *α* (stabilized by titanium) and phase *β* (stabilized by vanadium) can be seen. In general, the average roughness depends on the scanning scale, and for 25 × 25 µm^2^, S_a_ = 2.1 ± 0.6 nm for both treatments; and in the areas of 2 × 2 µm^2^, S_a_ = 0.15 ± 0.11 nm and 1.2 ± 0.8 nm in protocols *B* and *C*, respectively. These slight changes in the small-scale roughness are related to the increase in the number of groove-lines in the samples from treatment *C*, which must be related to rubbing the surface with cotton. When comparing the 25 × 25 images of the two passivation protocols, it was observed that small deposits appeared on the protocol *B* surface (small white dots in Figure 1a) that disappeared in the image associated with protocol *C*(Figure 1c). This fact is in agreement with the idea that protocol *C* can remove some polishing debris and could be responsible for the smaller deviations observed in the contact angle measurements.

As for the surface tension of Ti6Al4V ELI, it depends largely on the specific conditions of the passivation process and oxide layer formation on the studied surface. There are significant differences between the surface tension value (51.34 mN/m) for Ti6Al4V ELI prepared according to procedure *A* (spontaneous passivation on air) and that for Ti6Al4V ELI prepared according to procedure *B* (34.91 mN/m) or *C* (35.84 mN/m). In the case of Ti6Al4V ELI’s surface after passivation in the air, its surface tension and its components and parameters are higher than before. This indicates that after the passivation of Ti6Al4V ELI in the air, its surface becomes more hydrophilic. The specific conditions of surface preparation particularly influence the $\gamma^{AB}$ component and electron donor ($\gamma^{-}$) parameter of Ti6Al4V ELI’s surface tension values (Table 3). The changes in the Lifshitz–van der Waals component of Ti6Al4V ELI’s surface tension ($\gamma^{LW}$) are also significant. The changes prove also that the TiO_2_ layer on Ti6Al4V ELI’s surface formed in the water environment is more homogeneous.

Table 3 shows that rubbing with a cotton material before the cleaning procedure influences the $\gamma^{-}$ value. This probably results from the fact that rubbing with cotton removes some of the SiO_2_ particles from Ti6Al4V ELI after polishing. The SiO_2_ particles are present in the polishing liquid used in the last step of Ti6Al4V ELI’s mechanical polishing procedure. The surface cotton rubbing step of surface preparation influences the standard deviations of the measured contact angle values of model liquids (especially water) too, which probably results from the fact that the oxide layer formed on Ti6Al4V ELI’s surface prepared according to procedure *C* is more homogeneous compared to that formed on the surface prepared according to procedure *B*. It could influence the adsorption of water molecules in the cracks of oxide layer formed on Ti6Al4V ELI’s surface. From the data presented in Table 3 it can also be stated that the changes in the values of components and parameters of model liquids surface tension influence only the $\gamma^{-}$ values of Ti6Al4V ELI’s surface tension.

As was stated earlier, for the calculations of components and parameters of the solid’s surface tension using a given method, data of results from the same method should be used [14,27,29,30]. Thus, for further considerations of the wettability process in the studied system, Ti6Al4V ELI’s surface tension and its components and parameters, calculated from the model liquids’ surface tension values (and components and parameters), determined only on the basis of the contact angle measurements (1), were used.

### 2.2. Ti6Al4V ELI Wettability by the Aqueous Solutions of Sucrose Fatty Acid Esters

#### 2.2.1. Contact Angle Measurements

As stated earlier, the most important properties of titanium alloys are closely related to its practical applications as implants, including wear and corrosion resistance. For these reasons, Ti6Al4V ELI’s surface prepared according to procedure *B* was taken into account in the Ti6Al4V ELI wettability considerations. This was necessary because the presence of the oxide layer on Ti6Al4V ELI’s surface affects the corrosion resistance of the studied biomaterial. Thus, the particular activities in the surface preparation procedure should not influence on the properties of the passive layer. It should be noted that the oxide layer is formed only when the biomaterial is in contact with the air. In addition, it should be remembered that the oxide layer formed on the Ti6Al4V surface is characterized by poor mechanical properties, and it is easily fractured under fretting and sliding wear conditions. Oxide film distribution causes dissolution of the underlying metal [7].

**Figure 2:**
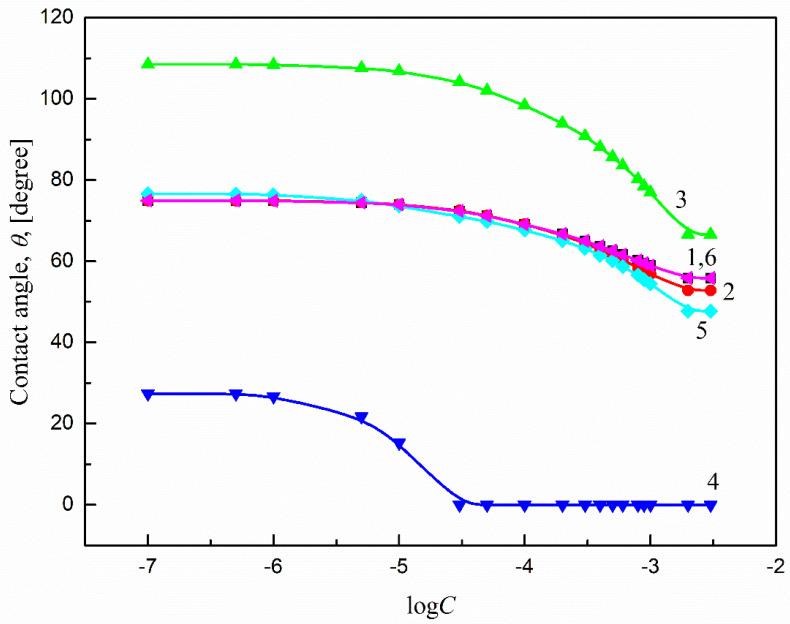
A plot of the contact angles (*θ*) of the aqueous solutions of SMD on Ti6Al4V ELI. Curves 1 and 2—the *θ* values calculated from Equations (6) and (5), respectively; curves 3 and 4—the *θ* values calculated from Equation (3) assuming the orientations of tail (curve 3) and head (curve 4) of the surfactant towards the solid–water; curve 5—the *θ* values calculated from Equation (7) and curve 6—the measured *θ* values.

**Figure 3:**
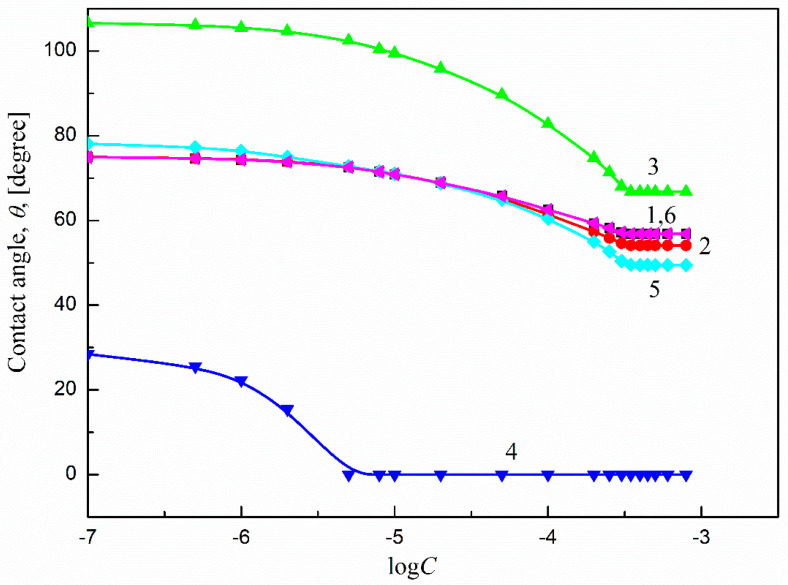
A plot of the contact angles (*θ*) of the aqueous solutions of SML on Ti6Al4V ELI. Curves 1 and 2—the *θ* values calculated from Equations (6) and (5), respectively; curves 3 and 4—the *θ* values calculated from Equation (3) assuming the orientations of tail (curve 3) and head (curve 4) of the surfactant towards the solid–water interface; curve 5—the *θ* values calculated from Equation (7) and curve 6—the measured *θ* values.

To study Ti6Al4V ELI’s wettability process, the contact angles (*θ*) of aqueous solutions of sucrose monodecanoate (SMD) and sucrose monolaurate (SML) at 293 K must be known. The measured values of *θ* in the studied system are presented in Figure 2 (curve 6) and Figure 3 (curve 6) as relations between *θ* and the respective logarithms of surfactant concentration in the solution. According to Equation (1), for the above-mentioned considerations, besides the liquid contact angle values, the properties of both the solid and the liquid must be known. Thus, for this purpose the values of surface tension for the aqueous solutions of SMD and SML at 293 K were taken into account. These values were measured and presented in earlier studies [33]. On the other hand, the components and parameters of Ti6Al4V ELI’s surface tension were determined from the components and parameters of the model liquids’ surface tension calculated based only on the contact angle values.

As shown in Figure 2 and Figure 3, the wettability of Ti6Al4V ELI in the studied systems depends on the type of surfactant and its concentration. It can be stated that the isotherms of contact angle are similar to those of surface tension [33]. There are also characteristic break points which relate to the critical micelle concentration values of the studied surfactants [33,34]. Past those points, the contact angles of the studied surfactant solutions are practically stable. The minimal contact angle values are comparable and equal to 56° and 57° for SMD and SML, respectively (Figure 2 and Figure 3). As follows from these values, there was no complete wetting of Ti6Al4V ELI′s surface by the aqueous solutions of SMD or SML, even at surfactant concentrations equal to or higher than their critical micelle concentrations. Assuming that the surface tension of Ti6Al4V ELI does not change during its wetting by the aqueous solutions of SMD and SML, it can be stated that the contact angle value of the studied solutions depends only on the solid surface tension and the solid–water interface tension (Equation (1)).

It should be also remembered that if during the wettability process of a given solid, surfactant molecules are able to penetrate into the solid surface and due to this fact change its surface tension, then Equation (3) can be written as follows:

(5) $$\gamma_{L}\left(\cos\theta +1\right)=2\left(\sqrt{\gamma_{L}^{LW}\gamma_{S}^{LW}}+\sqrt{\gamma_{L}^{+}\gamma_{S}^{-}}+\sqrt{\gamma_{L}^{-}\gamma_{S}^{+}}\right)-\pi_{e}\;$$

where the $\pi_{e}$ is the surfactant film pressure which can be determined from the Neuman et al. equation [24,35].

Using the Neumann et al. equation [24,35], Ti6Al4V ELI’s surface tension changes as a result of SMD and SML adsorption were determined. It was found that the surface tension of Ti6Al4V ELI is not stable during the wettability process, and changes take place with both SMD and SML. Moreover, the values of SMD and SML film pressure depend on the surfactant concentration in the solution.

From the practical point of view, it was particularly important to find out if it is possible to predict the contact angle values on Ti6Al4V ELI’s surface, as has been done earlier for different materials [14,27,36]. Due to the fact that Ti6Al4V ELI’s surface tension is reduced by the surfactant film formation around the solution drop settled on the surface, it is impossible to predict the surfactant solution’s contact angle value on the basis of Equation (4). In such cases, the values of the film pressure must be taken into account (Equation (5)). However, it should also be remembered that the solid surface tension reduction depends on the surfactant molecules’ orientation towards the solid–water interface. This orientation can be perpendicular (by head or by tail) or parallel. In the case of the perpendicular orientation of the surfactant molecules, the surface tension of the solid with the surfactant film depends on the tail or head predominance for the surfactant surface tension and its components and parameters. On the other hand, in the case of parallel orientation, the surface tension between the solid surface and the surfactant film is equal to the average of the tail and head orientations’ values. Thus, the maximal difference between the solid surface tension and that of the solid with the surfactant film is equal to $\frac{\pi_{e}}{2}\;$, and Equation (5) can be written as follows [14]:

(6) $$\gamma_{L}\left(\cos\theta +1\right)=2\left(\sqrt{\gamma_{L}^{LW}\gamma_{S}^{LW}}+\sqrt{\gamma_{L}^{+}\gamma_{S}^{-}}+\sqrt{\gamma_{L}^{-}\gamma_{S}^{+}}\right)-\frac{\pi_{e}}{2}$$

Thus, the values of the theoretical contact angle of the aqueous solutions of SMD and SML on Ti6Al4V ELI’s surface were calculated according to Equations (4) and (5). For this purpose, the values of components and parameters of Ti alloy surface tension determined from those of components and parameters of model liquids based on the contact angle were taken into account (Table 3). The values of the aqueous solutions of SMD or SML surface tension were taken from our previous paper [33]. The contact angles calculated from Equations (5) and (6) are presented in Figure 2 (curves 1 and 2, respectively) and Figure 3 (curves 1 and 2, respectively). The best fit between the measured and calculated contact angle values of SMD and SML aqueous solutions was found when Equation (6) was used (for both SMD and SML). The measured and predicted contact angle values are almost the same. This also proves that the SMD and SML molecules are parallel oriented at the Ti6Al4V ELI–water interface. The same conclusion was also drawn when the contact angle values were calculated while assuming that the surfactant molecules were perpendicularly oriented (by tail or head) (Figure 2 (curves 3 and 4, respectively) and Figure 3 (curves 3 and 4, respectively)) at the solid–water interface. There was no correlation between the measured and calculated contact angle values, even in the surfactant concentration range corresponding to its unsaturated monolayer at the water–air interface [33]. The parallel orientation of surfactant molecules at the solid–water interface can be also proved using the Baxter and Cassie equation [37,38]:

(7) $$\cos\theta =x_{1}\cos\theta_{1}+x_{2}\cos\theta_{2}$$

where $\theta_{1}$ and $\theta_{2}$ are the contact angles of the surfactant solution assuming the tail and head orientation towards the solid–water interface and that $x_{1}$ and $x_{2}$ are the surfactant’s contactable areas at the tail and head. The values of $x_{1}$ and $x_{2}$ were taken from the literature [39]. The obtained results are presented in Figs. 2 and 3 (curve 5). The contact angle values calculated from Equation (7) prove the parallel orientation of SMD and SML molecules at the Ti6Al4V ELI–water interface. There is also quite good agreement (especially in the case of SMD) between the measured contact angle values (Figure 2 (curve 6) and Figure 3 (curve 6)) and those calculated using Equation (7) for the range of surfactant concentration corresponding to having an unsaturated monolayer at the water–air interface. Thus, considering the measured and calculated contact angle values of the aqueous solutions of the studied surfactants at the Ti6Al4V ELI–water interface and those determined earlier [14] for the polymeric solids, it can be stated that the orientation of SMD and SML molecules towards the solid–water interface depends on the type of solid, and in the case of polar ones (PMMA, nylon 6, Ti6Al4V ELI) is parallel.

#### 2.2.2. Surface Excess Concentrations of SMD and SML at the Ti6Al4V ELI-Water Interface

The orientation of SMD and SML molecules influences their quantities at the Ti6Al4V ELI–water interface. The surface excess concentration ($\Gamma_{SL}^{}$) can be directly calculated from the Gibbs isotherm adsorption equation [17]:

(8) $$\Gamma_{SL}^{}=-\frac{C_{S}}{nRT}{\left(\frac{\partial \gamma_{SL}}{\partial C_{S}}\right)}_{T}=-\frac{1}{nRT}{\left(\frac{\partial \gamma_{SL}}{\partial \ln C_{S}}\right)}_{T}=-\frac{1}{2.303nRT}{\left(\frac{\partial \gamma_{SL}}{\partial \log C_{S}}\right)}_{T}$$

where *Cs* is the surfactant concentration in the solution; $\gamma_{SL}$ is the solid–water interfacial tension; *n* is the number depending on the kind of surfactant, which was assumed to be equal to 1 for the nonionic ones; R is the gas constant; and T is the temperature.

**Figure 4:**
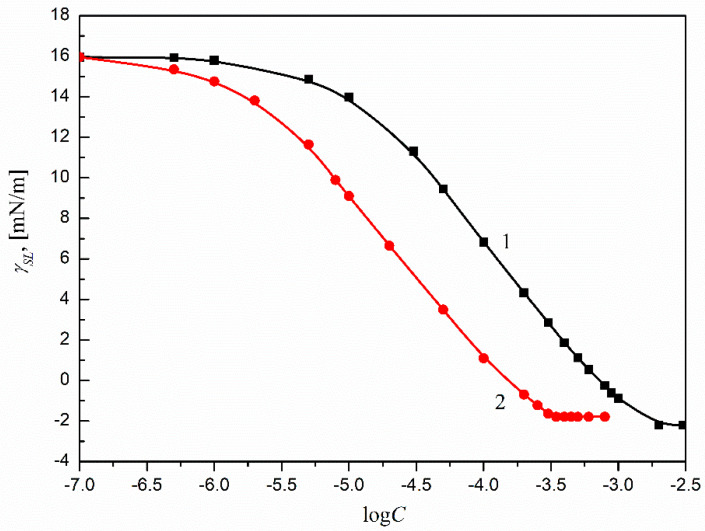
A plot of the Ti6Al4V ELI–water interface tension ($\gamma_{SL}$) of the aqueous solutions of SMD (curve 1) and SML (curve 2) vs. the logarithms of surfactant concentration (log*C*_S_).

For the $\Gamma_{SL}^{}$ calculations, the changes of $\gamma_{SL}$ in the whole range of the solution surfactant concentration must be known. According to the earlier, considerations the $\gamma_{SL}$ values were calculated based on Equation (1) and assuming that a surfactant film is formed around the solution drop settled on Ti6Al4V ELI’s surface. The results are presented in Figure 4, which shows that the values of $\gamma_{SL}$ depend on the type of surfactant and its concentration in the solution. Moreover, the $\gamma_{SL}$ changes with the surfactant concentration in the solution for both SMD and SML can be described by a second-order exponential function, which was next used for the $\Gamma_{SL}^{}$ calculations.

**Figure 5:**
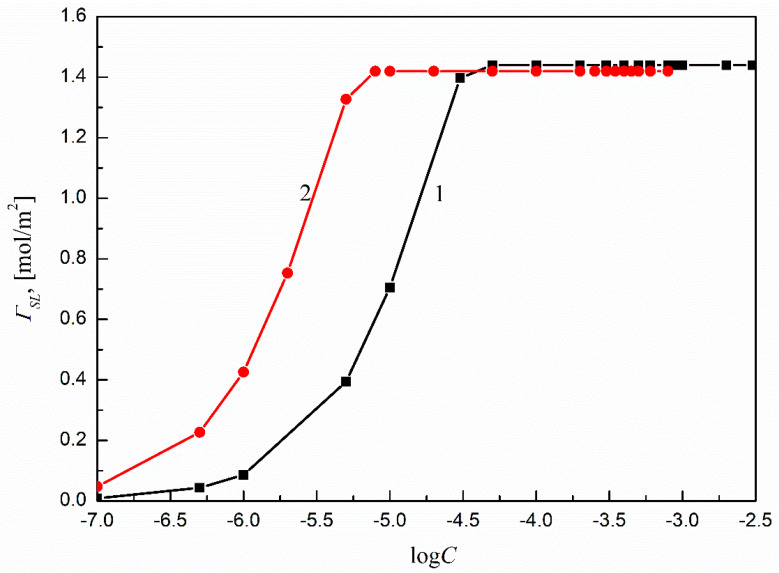
A plot of the SMD (curve 1) and SML (curve 2) Gibbs surface excess concentrations ($\Gamma_{SL}^{}$) at the Ti6Al4V ELI–water interface vs. the logarithms of surfactant concentration (log*C*_S_).

The calculated $\Gamma_{SL}^{}$ values are presented in Figure 5. The maximal values of $\Gamma_{SL}^{\max}$ for SMD and SML were determined from their respective surfactant concentration ranges corresponding to linear dependence between $\gamma_{SL}$ and log*Cs*, which also reflects the saturated character of the solid–water interface. The $\Gamma_{SL}^{\max}$, or the minimal area of surfactant molecules at the solid–water interface ($A_{S}^{\min}$) [17], reflects the orientation of the surfactant molecules at the solid–water interface. By our calculations, the SMD and SML molecules were parallel at the solid–water interface, and the maximal amounts of surfactant at the Ti6Al4V ELI–water interface were practically the same as in the case of PMMA [14], equal to 1.44 and 1.42 mol/m^2^ for SMD and SML, respectively. These values are also close to those calculated theoretically [14].

**Figure 6:**
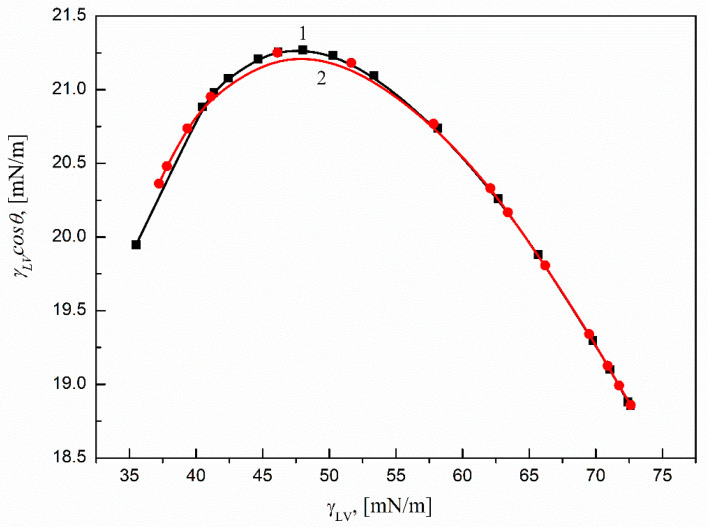
A plot of the adhesion tension ($\gamma_{LV}\cos$) of the aqueous solutions of SMD (curve 1) and SML (curve 2) vs. the solution surface tension $\gamma_{LV}$.

The relative amount of the surfactant at the solid–water interface can be also calculated by means of the Lucassen–Reynders equation [17]. For that, the dependence between the adhesion tension ($\gamma_{LV}\cos\theta$) and the surface tension ($\gamma_{LV}$) of the surfactant solution can be used. To determine the relative amount of surfactant in the studied range of concentration, this relation should be linear. Thus, in our studies the relationships between $\gamma_{LV}\cos\theta$ and $\gamma_{LV}$ for SMD and SML were determined, and obtained results are presented in Figure 6.

.

As follows from this figure it is not possible to describe the above-mentioned relation of the Ti6Al4V ELI–surfactant (SMD or SML)–air system with one linear function in the whole studied surfactant concentration range. It can be noticed that for both SMD and SML the relation between $\gamma_{LV}\cos\theta$ and $\gamma_{LV}$ can be divided into two parts. The first part refers to the surfactant concentration range corresponding to the unsaturated monolayer of surfactant at the water–air interface, and the second one refers to the saturated monolayer at this interface. Thus, based on these two relations, it was possible to determine only the so-called critical surface tension of solid wetting ($\gamma_{C}$), which describes the surface tension of a liquid at which the complete wetting of a given solid takes place [17]. It turns out that the $\gamma_{C}$ of the Ti6Al4V ELI–surfactant depends on the kind of surfactant used for its determination, and in the studied system it depends also on the surfactant′s concentration in the solution. For SMD and SML, the concentrations corresponding to an unsaturated monolayer of surfactant at the water–air interface $\gamma_{C}$ were equal to 25.59 mN/m and 25.47 mN/m, respectively. The obtained $\gamma_{C}$ values were quite different from those determined for the SMD and SML concentrations corresponding to the saturated monolayer at the water–air interface (16.51 mN/m and 17.24 mN/m respectively). The obtained values are much smaller than Ti6Al4V ELI′s surface tension and close to the Lifshitz–van der Waals component of water′s surface tension (26.85 mN/m) [29]. These values indicate also that Ti6Al4V ELI′s surface properties change during the wettability process due to the studied surfactants’ adsorption at the solid–water interface. They also show that the SMD and SML molecules’ orientations at the Ti6Al4V ELI–water interface depend on their concentrations in the solution. Furthermore, for both surfactant concentrations (corresponding to the unsaturated and saturated surfactant monolayers at the water–air interface), the $\gamma_{C}$ values of Ti6Al4V ELI wetting determined for SML were somewhat higher. That could have resulted from the better efficiency of SML adsorption at the Ti6Al4V ELI–water interface compared to SMD. According to the above-mentioned statement, the values of standard Gibbs free energy of SMD and SML adsorption at the Ti6Al4V ELI–water interface were calculated.

#### 2.2.3. Standard Gibbs Free Energy of SMD and SML Adsorption at the Ti6Al4V ELI–Water Interface

There are many approaches which can be used for determination of $\Delta G_{ads}^{0}$ [17,40,41,42,43,44,45]. One option is using the Langmuir equation modified by de Boer [17,42]:

(9) $$\frac{A_{SL}^{0}}{A_{SL}-A_{SL}^{0}}\exp\frac{A_{SL}^{0}}{A_{SL}-A_{SL}^{0}}=\frac{C_{S}}{\omega }\exp\left(\frac{\Delta G_{ads}^{0}}{RT}\right)$$

where $A_{SL}^{0}$ is the area occupied by the surfactant molecule at the solid–water interface, ω is the number of water moles in 1 dm^3^. To calculate $\Delta G_{ads}^{0}$ from Equation (9) the values of $A_{SL}^{0}$ for a given surfactant must be known. Among other things, the $A_{SL}^{0}$ values can be determined from the Joos equation of state, which for the aqueous solutions of surfactants can be written in the following form [46]:

(10) $$\exp\left(\frac{-\pi }{RT\Gamma_{W}^{\infty }}\right)+\exp\left(\frac{-\pi }{RT\Gamma_{SL}^{\infty }}\right)\frac{C_{S}}{a_{SL}^{s}}=1$$

where $\Gamma_{SL}^{\infty }$ is the limiting Gibbs surface excess concentration of water at the solid–water interface, π is the film pressure and $a_{S}^{s}$ is the activity of a given surfactant at the solid–water interface.

The $\Gamma_{SL}^{\infty }$ and $A_{SL}^{0}$ values for SMD and SML in the studied system were equal to 63.5 and 69 Å^2^, respectively. These values are similar to those calculated theoretically from the literature (60.93 Å^2^) [14,47].

**Figure 7:**
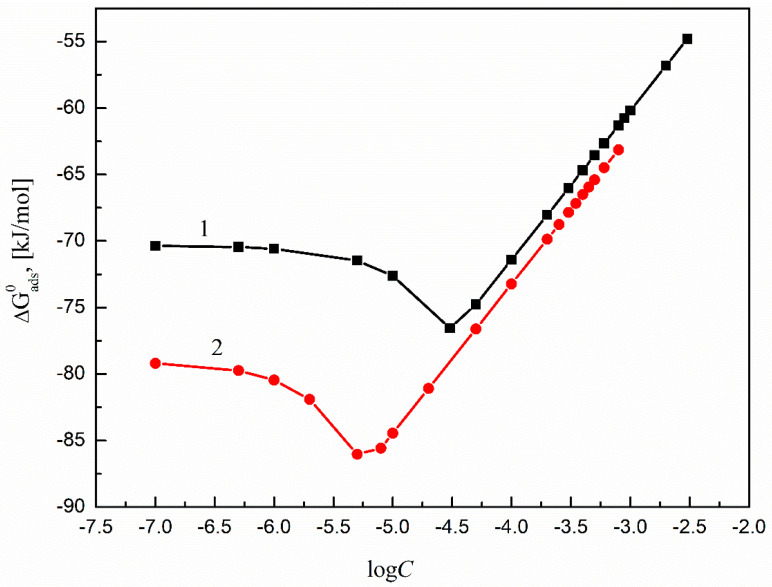
A plot of the standard Gibbs free energies of adsorption ($\Delta G_{ads}^{0}$) of SMD (curve 1) and SML (curve 2) at the Ti6Al4V ELI–water interface vs. the logarithms of surfactant concentration (log*C*_S_) calculated from Equation (9).

The calculated $\Delta G_{ads}^{0}$ values of all studied surfactants are presented in Figure 7. The values for SML are somewhat smaller than those for SMD and prove better efficiency of SML adsorption at the Ti6Al4V ELI–water interface compared to SMD.

#### 2.2.4. Work of Adhesion of the Aqueous SMD and SML Solutions to the Ti6Al4V ELI Surface

From the practical point of view, it is very important to predict the ability of a given substance to cover a solid surface. The process is especially desirable in the systems that include polymers and metallic implants. For example, in systems containing titanium alloys, the surface coating influences the corrosion resistance of the materials. In our studies, due to their adsorption and bacteriostatic properties, sucrose fatty acid esters were used to cover Ti6Al4V ELI’s surface. As surfactants, their ability to coat a given solid surface can be estimated and predicted on the basis of the work of adhesion of the aqueous surfactant solution to the solid surface, which can be expressed as follows [17]:

(11) $$W_{A}=\gamma_{LV}+\gamma_{SV}-\gamma_{SL}$$

If $\gamma_{SV}$ and $\gamma_{SL}$ are not known and the contact angle (θ) of the liquid on the solid surface is larger than or strictly equal to zero, then the $W_{A}$ can be calculated from the following equation [17]:

(12) $$W_{A}=\gamma_{LV}\left(\cos\theta +1\right)$$

Additionally, if components and parameters of the liquid and the solid surface tension are known, $W_{A}$ can be generally calculated based on Equation (3), Equation (4) or Equation (5).

Considering the above-mentioned statements, the $W_{A}$ of the aqueous solutions of SMD and SML to Ti6Al4V ELI’s surface were calculated based on Equations (11) and (6), where the presence of surfactant film on the solid surface was taken into account.

**Figure 8:**
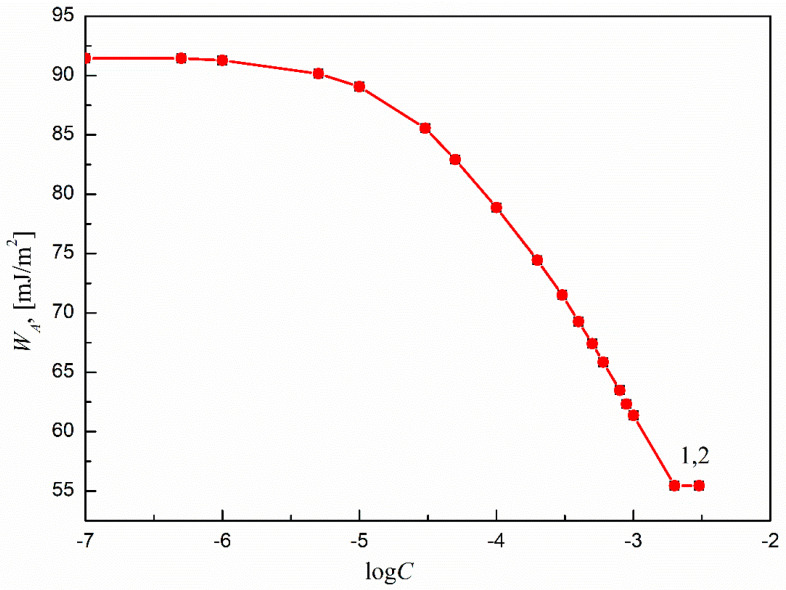
A plot of the work of adhesion ($W_{A}$) of the aqueous solutions of SMD to Ti6Al4V ELI’s surface vs. the logarithm of surfactant concentration (log*C*_S_). Curve 1 corresponds to the $W_{A}$ values calculated from Equation (9).

**Figure 9:**
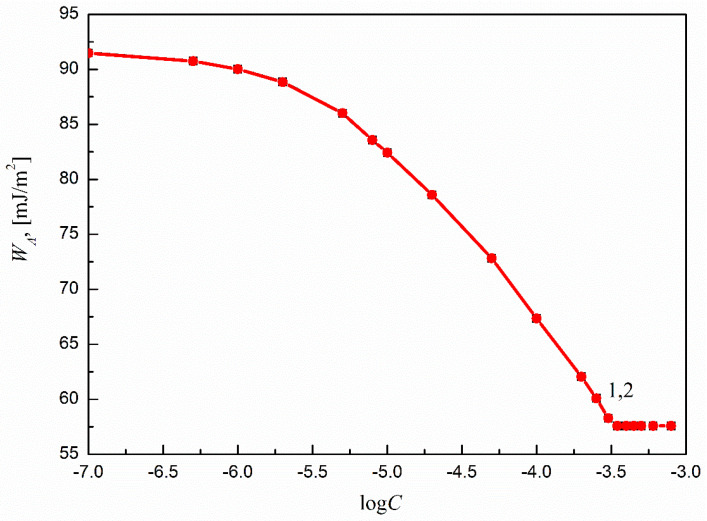
A plot of the work of adhesion ($W_{A}$) of the aqueous solutions of SML to Ti6Al4V ELI’s surface vs. the logarithm of surfactant concentration (log*C*_S_). Curve 1 corresponds to the *W_A_* values calculated from Equation (9).

The obtained results are presented and compared in Figure 8 and Figure 9. It can be seen that the $W_{A}$ of the aqueous solutions of both SMD and SML to Ti6Al4V ELI’s surface changes with the surfactant concentration in the solution. In addition, there is good agreement between the $W_{A}$ values calculated from Equations (11) and (6). This also indicates that in the case of Ti6Al4V ELI, similarly to other materials, it is possible to predict the $W_{A}$ of the aqueous solutions of surfactants to the studied solid. This is particularly important in the case of the systems that include biomaterials. Taking into account that both solid surface tension and $W_{A}$ for Ti6Al4V ELI change with the concentration of the surfactant in the solution, it can be stated that the changes are strictly related to the surface coverage by the studied surfactant molecules and the presence of the adsorption layer at the solid–water interface.

## 3. Conclusions

From the Ti6Al4V ELI wettability considerations it can be stated that:

The passivation of Ti6Al4V ELI under water has an influence on its surface properties. It caused significant increases in the contact angle values of all studied model liquids on Ti6Al4V ELI’s surface. This indicates that Ti6Al4V ELI’s surface after the underwater passivation becomes more hydrophobic. Additionally, the standard deviations of the measured contact angle values of the model liquids on Ti6Al4V ELI’s surface depend slightly on the kind of model liquid and Ti6Al4V ELI’s surface preparation procedure. The obtained results indicate that surface treatment of Ti6Al4V ELI according to procedure *C* causes the oxide passivation layer formed on the surface to be more homogeneous. On the other hand, the adsorption of any of the studied surfactants at the Ti6Al4V ELI–water interface caused a significant decrease in Ti6Al4V ELI’s surface tension.

Ti6Al4V ELI’s surface properties also change with the surfactant concentration in the solution. Assuming parallel orientation of the surfactant molecules at the solid–water interface and the presence of a surfactant film around the drop settled on the Ti6Al4V ELI, it was possible to predict the wettability of Ti6Al4V ELI by the aqueous solutions of SMD and SML for their whole concentration ranges. Moreover, the $W_{A}$ of the aqueous solutions of SMD and SML to Ti6Al4V ELI could be predicted. It was deduced that it changes with the studied surfactant concentration in the solution.

## 4. Experimental

### 4.1. Materials and Methods

Water (W), formamide (F) and diiodomethane (D) and aqueous solutions of sucrose monodecanoate (SMD) (purity > 97%) and sucrose monolaurate (SML) (purity > 97%) were used for the contact angle measurements. All chemicals were purchased from Sigma-Aldrich (Poland) and used without further purification. The aqueous solutions of the studied surfactants were prepared using ultrapure water (Milli-Q Plus, Darmstadt, Germany). The purity of water was additionally controlled by the surface tension and contact angle measurements before preparing the solutions. The concentration of SMD and SML ranged from 1 × 10^−7^ M to 3 × 10^−3^ M and from 1 × 10^−7^ M to 8 × 10^−4^ M, respectively.

### 4.2. Contact Angle Measurements

The measurements of the advancing contact angles of water, formamide and diiodomethane and the aqueous solutions of SMD and SML on Ti6Al4V ELI’s surface were acquired using the sessile drop method and the DSA measuring system (Krüss, Hamburg, Germany) in a thermostated chamber at 293.0 ± 0.1 K. The apparatus chamber was saturated with the vapor of a given liquid for which the contact angle was measured. The contact angle for a given solution was measured for at least 10 drops. The volume of the measured liquid drop was equal to 2 µL.

The commercially available Ti6Al4V ELI samples (Wolften, Wrocław, Poland) were used. Discs of 25 mm diameter and 2 mm in thickness were cut from a single bar by the manufacturer. Before use, the Ti6Al4V ELI samples were mechanically polished to the mirror standard (Grinder-Polisher, Buehler, Leinfelden-Echterdingen, Germany). For this purpose, the samples were abraded using silicon carbide paper and polished with diamond paste. Cleaning was finished using colloidal silica. All the materials used for the polishing of titanium discs were supplied by Buehler, Germany.

In procedure *A* of Ti6Al4V ELI’s surface preparation, the freshly polished Ti6Al4V ELI discs were carefully cleaned with distilled and next deionized water, and then ultrasonically cleaned for 10 min in water, acetone and ethanol. Acetone (purity > 99%) and ethanol (purity 96%) used for the purification of Ti alloy samples were bought from Sigma-Aldrich (Poland). After cleaning, the samples were dried in the oven for 1 or 2 h at 393 K. Then the samples were placed in a desiccator for 24 h or 48 h.

In procedure *B*, after polishing the samples were also carefully cleaned under the stream of distilled and next deionized water, and then ultrasonically cleaned for 10 min in water, acetone and ethanol. In the next step of the surface preparation procedure, the titanium alloy discs were put into the Milli-Q water for 24 h in the oven at 323 K. Next the discs were removed from water, dried in the oven for 1 or 2 h at 393 K and deposited in a desiccator for 24 h (or 48 h). In procedures *A* and *B*, some samples were dried in the oven at 393 K for 2 or 3 h to check if drying time affects the contact angle values. This procedure was used in another study [31].

In procedure *C*, the samples of Ti6Al4V ELI were carefully cleaned under the stream of distilled water after polishing, and the rubbed with cotton under a stream of warm distilled and next deionized water (the temperature of the water was equal to 333 K). After that step, the titanium alloy discs were cleaned, passivated and dried according to procedure *B*.

### 4.3. Characterization of Ti6Al4V ELI Properties

#### 4.3.1. XRF Analysis

The chemical composition analysis of Ti6Al4V ELI was performed using the WDXRF technique (S8 Tiger Bruker, Karlsruhe, Germany). The direct measurements were acquired in a He atmosphere. The analysis method was Quant Express. The mask diameter was equal to 28 m. The crystals used for the measurements (and their energy intervals and measurable elements) were: XS-5S (0.48–1.63 keV, from O to Al), PET (1.69–3.25 keV, from Si to Ar) and LiF (200) (3.25–58 keV, K to U). The results of the analysis are presented in Table 1.

#### 4.3.2. Atomic Force Microscopy (AFM) Analysis

An atomic force microscope (Agilent AFM 5500, Agilent Technologies, California, CA, USA) was used to estimate the surface topography of Ti6Al4V ELI prepared for the contact angle measurements. Images were obtained in the contact mode using tips with a nominal force constant 0.05 N/m and resonant frequency 14 kHz (CSC38AlBS, MikroMasch, Wetzlar, Germany). The Gwyddion software was used for further work with the images, and thus the average surface roughness (S_a_) was obtained.

## Figures and Tables

**Table 1:** Chemical analysis (%) of Ti6Al4V alloy.

Element	Ti6Al4V ELI	Manufacturer Data
Ti	89.940	Balance: about 90.0
Al	5.940	5.5–6.75
V	3.830	3.5–4.5
Fe	0.154	0.25 (max)

**Table 2:** The average values of the contact angle (*θ*) of water, formamide and diiodomethane measured at 293 K on Ti6Al4V ELI surface prepared according to different procedures.

Liquid	Spontaneous Passivation (Procedure *A*)	Procedure *B*	Procedure *C*
	*θ* [Degree]	*θ* [Degree]	*θ* [Degree]
Water	43 ± 5	75 ± 5	81 ± 2
Formamide	28 ± 5	61 ± 5	63 ± 2
Diiodomethane	39.0 ± 1.5	50 ± 3	49.0 ± 1.5

**Table 3:** The values of the Lifshitz–van der Waals ($\gamma^{LW}$), electron-acceptor ($\gamma^{+}$ ) and electron-donor ($\gamma^{-}$ ) parameters of the acid-base ($\gamma^{AB}$ ) components of model liquids’ (water, formamide and diiodomethane) surface tension taken from the literature [19,20,21,22,29] and those of Ti6Al4V ELI prepared according to procedure *A*, *B* or *C* and calculated from Equation (3) at 293 K.

**Liquid/Ti_6_Al_4_V-ELI**	$\gamma^{LW}$ [mN/m]	$\gamma^{AB}$ [mN/m]	$\gamma^{+}$ [mN/m]	$\gamma^{-}$ [mN/m]	γ [mN/m]
Water (1)	26.85	45.95	22.975	22.975	72.80
Formamide (1)	39.00	19.00	3.67	24.61	58.00
Diiodomethane (1)	50.80	0.00	0.00	0.00	50.80
Ti6Al4V ELI (procedure *A*)	40.1 ± 0.7	11 ± 5	1 ± 1	28 ± 9	51 ± 6
Ti6Al4V ELI (procedure *B*)	34.3 ± 1.8	1 ± 5	0.01 ± 0.2	10 ± 8	35 ± 7
Ti6Al4V ELI (procedure *C*)	34.8 ± 0.8	1.0 ± 1.6	0.05 ± 0.2	5 ± 2	36 ± 2
Water (2)	21.80	51.00	25.50	25.50	72.80
Formamide (2)	39.00	19.00	2.28	39.60	58.00
Diiodomethane (2)	50.80	0.00	0.00	0.00	50.80
Ti6Al4V ELI (procedure *A*)	40.1 ± 0.7	12 ± 3	1.1 ± 0.6	31 ± 7	52 ± 4
Ti6Al4V ELI (procedure *B*)	34.3 ± 1.7	2 ± 3	0.04 ± 0.2	12 ± 6	36 ± 5
Ti6Al4V ELI (procedure *C*)	34.8 ± 0.8	1.1 ± 1.2	0.04 ± 0.09	7.2 ± 2.0	36 ± 2

1—The values of the liquid surface tension and its components and parameters determined on the basis of the contact angle measurements [29]. 2—The values of the liquid surface tension and its components and parameters determined from the liquid–liquid surface tension [19,20,21,22].

## Data Availability

Data is contained within the article.

## Sample Availability

Samples of the compounds are not available from the authors.
